# Transradial versus transfemoral approach for coronary angiography and angioplasty – A prospective, randomized comparison

**DOI:** 10.1186/s12872-016-0457-2

**Published:** 2017-01-11

**Authors:** Fayaz Ahmad Bhat, Khalid Hamid Changal, Hameed Raina, Nisar Ahmad Tramboo, Hilal Ahmad Rather

**Affiliations:** 1 Internal Medicine, Sher-i-Kashmir Institute of Medical Sciences, Srinagar, India; 2 Present affiliation: Mercy St. Vincent’s Medical Center, Toledo, OH USA

**Keywords:** Transradial, Transfemoral, Angiography

## Abstract

**Background:**

PCI has been done traditionally through transfemoral route. But now transradial and transbrachial routes are also coming up in practice. We compared transradial versus transfemoral routes for ease of operability, time for procedure, complications, and failure rates through a prospective study.

**Methods:**

Four hundred Patients admitted in department of cardiology for percutaneous interventions were enrolled in the study. 200 patients were assigned to each group randomly. A single team did all the procedures. Pre procedure, intra procedure and post procedure data of all the patients was collected, tabulated and analysed properly.

**Results:**

Access time (6.0 ± 1vs 4.2 ± 0.7; *P* =0.001); Fluoroscopy time and overall procedure time (29 ± 11.3 Vs. 27.3 ± 12.4 min) were more with trans radial than transfemoral route, respectively. The most common post procedure complication, ecchymosis was seen in 20.5% in transfemoral group compared to 12.5% in transradial group (*P* 0.031). Thrombophelibites (17.5 VS 8%, *P*0.004); Hematoma (14.5 Vs 0%, *P* 0.005); post procedure access bleed (7 VS 3%, *P* 0.039) were seen in transfemoral than transradial group, respectively. Failure rates were almost similar. None of our patients had post procedure myocardial infarction, stroke, acute renal failure and infections.

**Conclusion:**

Transradial approach of PCI is better than transfemoral route with respect to complications like bleeding, haematoma formation, thrombophelebites and ecchymosis is concerned. However access and fluoroscopic time is more with the former. We recommend the transradial route for PCI.

**Trial registration:**

Trial is retrospectively registered in ClinicalTrials.gov with the Identifier: NCT02983721, Date of registration is December 2, 2016.

## Background

Coronary artery disease has had high morbidity and mortality for a long time. To date percutaneous coronary angiography and percutaneous transluminal coronary angioplasty are standard diagnostic and therapeutic strategy for coronary artery disease respectively [1]. The common femoral artery has long been the access site for doing coronary angiography and angioplasty. Femoral artery has been the preferred site of access because of the larger size and the larger diagnostic and angioplasty guiding catheters being used lately. The profile of balloons used is an additional factor for the same. The use of better coronary hardware and development of newer anticoagulants has significantly increased the spectrum for interventions. Vascular access site bleeding is one of the most feared complications particularly with the use of anticoagulants and platelet glycoprotein inhibitors [2]. Percutaneous coronary intervention (PCI) can be performed by the following three routes: femoral, brachial or radial arteries. The vascular or bleeding risk associated with the femoral approach is reported upto 10% in some studies [2, 3]. Other complications include hematomas accompanied by significant blood loss, arterial pseudo aneurysms and arteriovenous fistulas. The transradial approach for coronary procedures is gaining fast acceptance. It was first introduced by Compeau [2] in 1989 for diagnostic coronary angiography and it was subsequently improved upon by by Kiemenji and Laarman [3] for percutaneous transluminal coronary angioplasty and stenting. The interest in the transradial approach is increasing due to decreased associated vascular complications, convenience for the patients, earlier discharge, shorter stay in the hospital and early ambulation [4–7]. Not only is it a safer technique, but it is also characterized by its high success rate, close to 90% in some populations [8]. Vascular complications are lesser in the transradial approach because of favourable anatomy, smaller size of the sheaths used and rapid hemostasis. The main complications for the approach are smaller radial artery that may not be accessed successfully and arterial occlusion post procedure. Radial artery is smaller in the Asian populations compared to West [9, 10]. Bleeding complications are lesser and easily controllable with the radial approach because of the easy compressibility of the radial artery. Another advantage is that no big nerves or veins are located in the vicinity of the artery making injury to such structures less likely. Also there are economic benefits to the approach as reflected by reduced hospital expenditures. Patients overwhelmingly prefer the transradial over the femoral approach [10, 11]. Although transradial approach has a lot of benefits, it has a longer learning curve for the operator making it more challenging. It also limits the devices which are used in interventions like temporary pacemakers, intra-aortic balloon pumps and larger devices for coronary interventions. Also it may not always be the best choice in some patients who may have an anomalous palmer arch not providing sufficient blood supply to the hand in case of occlusion of radial artery. Due to this reason several authors recommend assessment of adequacy of collateral blood flow from the ulnar artery using Allen’s test before performing the procedure. Entry site failure is also one of the complications [12, 13]. Vascular access site preference is thus a choice in centers based on tradition and expertise [5]. We at our hospital did a comprehensive prospective study on this topic. The purpose of this study was to assess and compare the feasibility, success and safety of Transradial approach (TRA) verses Transfemoral approach (TFA) for diagnostic and therapeutic coronary angiography and coronary interventions.

## Methods

This study was conducted in the tertiary care hospital Sher-i- Kashmir Institute of Medical sciences Srinagar (SKIMS) in the department of Cardiology. It was a prospective study. We enrolled 400 patients, 200 patients for Transfemoral and 200 for Transradial diagnostic coronary angiography and therapeutic coronary interventions. Patients were randomly allocated to either group using simple randomization with odd serials going into one arm and even in to another. Patients who had any of the exclusion criteria mentioned below were not included in the study.

This study included patients taken for primary PCI, elective diagnostic coronary study and elective angioplasties. Transradial access was performed only if modified Allen’s test was normal (Positive), suggesting the presence of adequate collateral circulation from the ulnar artery. Adjuvant glycoprotein IIb/IIIa inhibitors were used wherever needed. Patients with impaired renal function tests, lack of informed consent, severe sepsis, local site infection, previous contrast allergy, severe intrinsic/iatrogenic coagulopathy (INR > 2) were excluded from the study. Exclusion Criteria for transradial route included an abnormal modified Allen’s test. Exclusion Criteria for transfemoral route included peripheral vascular disease (Iliofemoral disease).

Primary outcome measures included adverse events like bleeding, access site, and non access site complications. Also hospital stay duration and time of doing procedure were used. TIMI major bleeds were defined as intracranial or >5 g/dl in hemoglobin or a hematocrit drop of 15%. TIMI minor bleeds were observed blood loss with a >3 g/dl drop in hemoglobin or a 10% decrease in hematocrit or no observed blood loss with a >4 g/dl or a drop by >12% in hematocrit. Access site complications included surgical repair or intervention on the access site, pseudoaneurysm treated conservatively, or a large hematoma (documented as >5 cm). Nonaccess site complications included coronary artery dissection, acute myocardial infarction, coronary perforation, transient ischemic attack or cerebrovascular accident, and death during the index hospitalization. Procedural failure was defined as a combined endpoint of access site crossover, failed coronary angiography, or failed target vessel revascularization, was also assessed. Patient radiation exposure was estimated by total fluoroscopy time. Secondary end points were other lesser common events like sepsis, thrombophelebites, embolization etc. Sample size calculation was done using the method given by Sugimoto [14].

Our preference was to use the right radial and right femoral routes as they are nearest to operator while facing cardiac monitors, in our hospital. For the radial approach, the wrist was sterilized and draped in usual fashion. Hyperextension over an arm board was done and skin over the puncture site was anesthetized with 2 – 3 ml of 1% lignocaine. A small scaled incision was performed 1 cm proximal to styloid process of radius where arterial pulse was best felt. The radial artery was punctured with a 21 G needle and 6 F sheath (Cardis, Terumo) were introduced into the artery, using Seldinger technique. All patients received verapamil (5 mg) to reduce radial artery spasm. Heparin (weight adjusted) was used only in PCIs to prevent artery occlusion and not in elective diagnostic coronary studies. Long 0.038 Terumo guide wire was used under fluoroscopic guidance. The catheter used for transradial approach were specially designed for transradial route such as Tiger catheter (Terumo) sized 6 F. Similarly in case of transfemoral approach our preference was to use right femoral route. The groin was prepared and draped and the site was punctured for femoral access after anesthetizing the skin with 2–4 ml of 1% lignocaine. Once the femoral puncture was done 6 F sheath of Cordis variety was introduced and 6 F Judkins, catheter was introduced and it was guided under fluoroscopic guidance through the aortic route. All the patients undergoing this procedure received injection heparin (weight adjusted). Procedural time was calculated separately. Once the procedure was completed the radial sheath was removed in the lab and pressure over the site was applied for 2 h. Manual compression was used for hemostasis with two trained medical assistants performing the compression. No vascular closure devices were used. Patients were transferred back to the ward, where the radial site and femoral site were clearly monitored for bleeding and other complications. All the procedures were performed by a single team headed by one operator in order to reduce operator bias. Those patients where we failed to gain radial artery access; procedure was crossed over to femoral route and for the same reason groin was kept prepared.

Statistical analysis was done on an MS Windows-based PC computer. The data were first keyed into a Microsoft Excel spreadsheet and then analyzed by Statistical Package for the Social Sciences (SPSS), Version 20.0, from SPSS incorporation Chicago IL. We used mean, standard deviation/standard error of mean, and percentage when appropriate for the patient’s characteristic description. Group differences were compared using the Pearson *χ*2 or Fisher's exact test for categorical variables, or the Student *t* test or the Mann–Whitney *U* test for continuous variables. *P*-values of 0.05 or less were considered statistically significant.

## Results

**Fig. 1:**
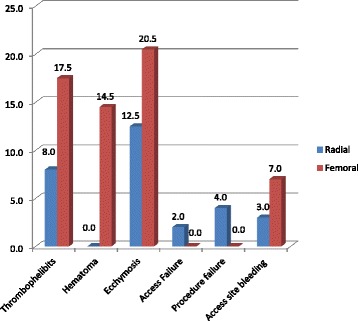
Comparison of complications in the studied subjects between the transradial and transfemoral group

A total of 400 patients were enrolled in this prospective comparative study over a period of 2 years and 2 months from September 2013 to November 2015 (200 patients in transradial approach group and 200 in transfemoral approach group). Table 1 shows the gender distribution, smoking habits, dwelling and body mass index with respect to each group (transradial or transfemoral). None of the patients had undergone a previous PERCUTANEOUS TRANSLUMINAL CORONARY ANGIOPLASTY. Table 2 shows the procedure characteristics of studied subjects. The commonest single vessel involved was left circumflex (LCX) (15% in transfemoral group VS 11.7% in transradial group). LAD + RCA (left anterior descending + right coronary artery) disease was common in transfemoral group and LCX + RCA disease was commonly picked in transradial group. Double vessel disease was commonly seen in both the studied arms. In transradial group 102 (51%) patients underwent PCI plus stenting, 30 (15%) patients underwent rescue PCI plus stenting, 68 (34%) patients underwent double vessel stenting. In transfemoral group 118 (59%) patients underwent PCI + Stenting, 25 (12.5%) underwent rescue PCI+ Stenting, and the double vessel stenting was done in 57 (28.5%) patients. Table 3 and Fig. 1 show the comparison for complications in the two groups.

**Table 1:** Demographic characteristics of the studied subjects

Variable		Radial		Femoral		*p* value
		*N* %		*N*	%	
Gender	Male	148 (74%)		134	67.0	0.279 (NS)
	Female	52 (26%)		66	33.0	
Dwelling	Rural	114 (57%)		112	62.0	0.472 (NS)
	Urban	68 (43.0%)		76	38.0	
Smoking History		160 (80%)		144 (77%)		0.607
Body Mass Index (Kg/m^2^) mean ± SD		27.1 ± 2.4		26.9 ± 1.6		0.665 (NS)
Age, Years	mean ± SD	61.8 ± 6.6		60.6 ± 10.0		0.31 (NS)
	≤50	16	8	38	19	
	51 to 60	78	39	36	18	
	61 to 70	88	44	102	51	

There were 148 males (74%) and 52 females (26%) in the transradial group and there were 134 (67%) males and 66 (33%) females in the transfemoral group. Both in transradial and transfemoral group rural populace outnumbered the urban dwellers. Most of the studied subjects were smokers. The mean BMI (body mass index) of (27.1 ± 2.4) and (26.9 ± 1.6) were observed in transradial and transfemoral group of studied subjects respectively. Most of our studied subjects were in the age group of 51 to 70 years in both the arms

*Abbreviations*: *N* number, *NS* not significant, *SD* Standard Deviation

**Table 2:** Showing procedure characteristics of studied subjects

		Radial	Femoral	*p* value
		*N* %	*N* %	
Type of case	Emergency	23 (11.5%)	31 (15.5%)	0.242 (NS)
	Elective	177 (88.5%)	169 (84.5%)	
Access time (min)		6.0 ± 1.8 (3, 10)	4.2 ± 0.7 (3, 5)	<0.0001 (Sig)
Fluoroscopy time (min)		6.4 ± 2.9 (3, 15)	6.0 ± 2.5 (3, 15)	0.015 (sig)
Procedure time (min)		29.0 ± 11.3 (12, 60)	27.3 ± 12.4 (12, 60)	0.03 (sig)
Crossover to femoral		8 (4%)	0	0.97 (NS)
Hospital Stay (days), Mean ± SD		3.6 ± 1.3	4.0 ± 1.1	0.009 (sig)

The site of access was right radial and right femoral respectively in the transradial and transfemoral group of patients. Most of the patients were electively selected. Access time was more in transradial group compared to transfemoral group of patients (6.0 ± 1.8 vs 4.2 ± 0.7 min) and it was statistically significant. The fluoroscopy time was more in transradial group compared to transfemoral group and the overall procedure time was also more in transradial group (29 ± 11.3 VS 27.3 ± 12.4 min). The mean hospital stay was 3.6 ± 1.3 days (with the range of 2 to 6 days) in the transradial group compared to 4.0 ± 1.1 days (with the range of 2 to 6 days) in transfemoral group of patients which was statistically significant

*Abbreviations*: *N* number, *NS* not significant, *sig* significant, *SD* standard deviation

**Table 3:** Comparison of complications in the studied subjects

Variable	Radial	Femoral	*p* value
	*N* (%)	*N* (%)	
Hematoma	0	29 (14.5%)	0.005 (Sig)
Bleeding complications	6 (3%)	14 (7%)	0.039 (sig)
TIMI major bleeding	0	8 (4%)	
TIMI minor bleeding	6 (3%)	4 (2%)	
Required Blood transfusion	0	2 (1%)	
Access site complications	0	10 (5%)	0.0003 (sig.)
Access site surgery/intervention	0	1 (2%)	0.63 (NS)
Thrombophlebitis	16 (8%)	35 (17.5%)	0.004 (Sig)
Pseudoaneurysm	0	2 (1%)	1.87 (NS)
Hematoma >5 cm	0	3 (1.5%)	0.002 (sig.)
Coronary perforation	0	1 (0.5%)	2.32 (NS)
Coronary dissection	0	1 (0.5%)	2.31 (NS)
Ecchymosis	25 (12.5%)	41 (20.5%)	0.031 (Sig)
Thrombosis of vessel	1 (0.5%)	0	1.000 (NS)
Access Failure	4 (2%)	0	0.082 (NS)
Sepsis	0	0	1.000 (NS)
Acute Renal injury	0	0	1.000 (NS)
Myocardial infarction	0	0	1.000 (NS)
Stroke	0	0	1.000 (NS)
Procedure failure	8 (4.0%)	0	<0.0001 (Sig)
Infective complications	0	0	1.000 (NS)
Death during hospitalization	0	1 (0.5%)	2.69 (NS)

The commonest post procedure complication was puncture site ecchymosis in 20.5% in transfemoral group compared to only 12.5% in transradial group which is statistically significant. 17.5% developed thrombophelebites in transfemoral group compared to only 8% in transradial group which is statistically significant. Hematoma developed in 14.5% in transfemoral group patients compared to none in transradial group which is statistically significant. Post procedure access site bleeding was seen in 3% patients in transradial group compared to 7% in transfemoral approach which was statistically significant. However there was more access failure rate in transradial group (2%) while as none had it in transfemoral group. Similarly the procedure failure rate was 4% (2% due to access failure 2% due to problem in guide wire hooking) in the transradial group compared to none in transfemoral group and it was statistically significant. One patient (0.5%) developed post procedure thrombosis of the vessel. None of our patients had post procedure Myocardial infarction, Stroke, Acute Renal injury and infections

*Abbreviations*: *N* Number, *Sig* significant, *NS* not significant

## Discussion

We performed therapeutic procedures like primary percutaneous coronary intervention (PCI), rescue PCI and elective stenting for Coronary artery diseases (CAD). Transradial approach via right radial artery was performed in 200 patients (148 males and 52 females) likewise transfemoral approach via right femoral artery was used in 200 patients (134 males and 66 females). The mean age of the patients in the transradial approach group was 61.8 ± 6.6 years and 60.6 ± 10 years was in transfemoral approach. Most of the patients in both the studied arms were in the age group of 51 to 70 years. This reiterates the fact that age is an important risk factor for CAD. The purpose to undertake this study was to assess and compare the feasibility, safety and success between the two approaches. The end result was to look for the access time, total procedure and total fluoroscopic time, procedure failure rate, complications associated with the procedures and total hospital stay in days in either of the procedures. In our study we found that the access time was more with the transradial approach compared to transfemoral approach (6.0 ± 1.8 min versus 4.2 ± 0.70 min, *p* value of <0.0001). The total procedure time was also more in transradial approach group compared to transfemoral approach group (29 ± 11.3 min versus 27.3 ± 12.4 min, *p* value of 0.03). Similarly the total fluoroscopic time was more in transradial approach compared to transfemoral approach (6.4 ± 2.9 min versus 6.0 ± 2.5 min *p* value 0.015). Similar results were found in the study conducted by Saleem Kassman et al. [15]. Ferdinand Kiemeneij et al. [16] also showed similar results in relation to procedure time and fluoroscopic time. Our results in terms of access time in two approaches were similar to the results shown in the study by Veli Vefali et al. [17] and to a meta-analysis on 12 trials [18]. The higher time requirements for the procedure in transradial group is usually due to radial artery spasm (can be prevented by using vasodilators), presence of tortuous subclavian arteries in many patients (causing obstacles in advancing the catheters), abnormal radial artery anatomy and operator dependent. In our study crossover from radial to femoral approach was required in 4% patients. Saleem Kassman et al. [15] in their study had crossover from radial to femoral route required in up to 4% as well. Similarly Ferdinand Kiemeneij et al. [16] in their study found that access failure was more common with transradial procedure. The failed attempts in transradial group are usually due to radial artery puncture failure, radial artery spasm, the size of the catheter used, type of procedure being done (diagnostic vs. therapeutic, which also affects the size of catheter used), tortuosity of the innominate trunk, dilatation of the ascending aorta, lusoria artery and inability to track the catheter in the left main coronary artery. Procedural success has also been shown to be higher in trans-femoral PCI in an updated report from the US national cardiovascular data registry as well [19]. In the RIVAL trial, radial and femoral approaches were both found to be safe and effective for PCI but lower rate of local vascular complications were seen in the radial approach [20].

In our study we found that post procedure complications were more common in the trans-femoral approach group compared to transradial group. The common complications were puncture site ecchymosis in 20.5% in transfemoral group compared to only 12.5% in transfemoral group (*p* < 0.05). Hematomas were seen in 14.5% of trans-femorals compared to none in the radial group. Thrombophelibites was more common in the transfemoral group (17.5%) compared to only (8%) in transradial group with *p* < 0.05. Bleeding complications were also seen in a significantly higher number of patients in the TFA compared to TRA. Also the TFA group had a higher number of more severe bleeding episodes. R. Choussatet al [21] in their study found that access site bleeding was seen in 7.4% in transfemoral [TFA] group where as none had hematoma formation in transradial [TRA] group [*p* = 0.04]. Agostoniet al [8] in their study found that TRA was associated with a significantly lower rate of complication, even at the cost of higher rate of procedure failure and the results were consistent with our study. Similarly Kassman et al. [15] in their study found that radial access was associated with low rate of access site related major bleeding (*p* = 0.04). Vefali et al. [17] in their study found that only minor complications were seen during transradial approach most commonly being pain ecchymosis. 5.4% developed hematoma at the access site in the transfemoral group and the results of our study were comparable with the above study. Bleeding complications were also significantly higher in the updated report from the US national cardiovascular data registry [19]. Major trials conducted for comparison of the two procedures showed similar conclusions. In the RIFLE-STEACS trial; a multicenter, randomized, parallel-group study, it was found that radial access in patients with ST-segment elevation acute coronary syndrome was associated with significant clinical benefits, in terms of both lower morbidity and cardiac mortality. Thus, it should become the recommended approach in these patients, provided adequate operator and center expertise is present [22]. Similarly, In the MATRIX access the authors demonstrated that in patients with non-ST-segment elevation acute coronary syndrome undergoing PCI, radial access was associated with significant reduction in major bleeding (OR 0.52, *p* 0.0002), access-site bleeding (OR 0.41, *p* 0.007), and need for blood transfusions (OR 0.61, *p* 0.02). Furthermore, the 1-year mortality was significantly lower in radial approach (OR 0.72, *p* 0.02) [23]. Thus the evidence points that trans-radial approach has significant clinical benefits than trans-femoral approach. In addition a study conducted on 203 patients undergoing TRA who had normal, intermediate, and abnormal Allen test results found safety and feasibility of TRA across the whole spectrum of Allen test results thus pointing to the safety of TRA even in patients with abnormal Allen tests [24]. Also, new evidence suggests that PCI with 7 F guiding catheter is feasible and safe [25].

In our study we found that the hospital stay was less in transradial approach group compared to transfemoral group (3.6 ± 1.3 days versus 4.0 ± 1.1 days, *p* = 0.009) and it was statistically significant. Choussatet al [20] in their study found that the total hospital stay was 5.0 ± 4.3 days in the transradial group versus 4.9 ± 4.3 days in transfemoral group which was to some extent contrary to our results. This could be due to the higher number of PCIs done at our center each year (>1000 per year) giving good expertise. However our results were consistent with the study by Vefali et al. [17] and Ferdinand Kie and also with the updated report from the US national cardiovascular data registry [19].

Important conclusions can be drawn from our study. Transradial approach has many potential advantages compared to transfemoral approach. These include decreased access site complications, decreased bleeding, early ambulation and shorter duration of hospital stay. Potential disadvantages of transradial approach compared to transfemoral approach include a longer learning curve for the operator, increased access failure and access site crossover, longer procedure, fluoroscopy time and greater radiation exposure in non-experts and possible lower procedure success rate in non-experts. Transradial approach is safer as compared to transfemoral approach because there are minimal post procedure vascular complications. However, transradial approach is more time consuming procedure. Overall hospital stay is less with transradial approach compared to transfemoral approach which is more needed in the developing countries like our country where there is a scarcity of the hospital beds and the recent increasing burden of coronary artery disease.

## Conclusion

Transradial approach of PCI is better than transfemoral route with respect to complications like bleeding, haematoma formation, thrombophelebites and ecchymosis is concerned. However access and fluoroscopic time is more with the former. We recommend that more emphasis should be put on rapid spread of expertise in trans-radial approach. Trans-radial approach may soon become the standard mode of access for PCIs and we recommend the same in the Indian subcontinent where we carried our study.

## Abbreviations

CAD: Coronary artery diseases
PCI: Percutaneous coronary intervention
SKIMS: Sher-i- Kashmir Institute of Medical sciences Srinagar
SPSS: Statistical Package for the Social Sciences
TFA: Transfemoral approach
TRA: Transradial approach
